# It’s about time: Linking dynamical systems with human neuroimaging to understand the brain

**DOI:** 10.1162/netn_a_00230

**Published:** 2022-10-01

**Authors:** Yohan J. John, Kayle S. Sawyer, Karthik Srinivasan, Eli J. Müller, Brandon R. Munn, James M. Shine

**Affiliations:** Neural Systems Laboratory, Department of Health Sciences, Boston University, Boston, MA, USA; Departments of Anatomy and Neurobiology, Boston University, Boston University, Boston, MA, USA; Department of Radiology, Massachusetts General Hospital, Boston, MA, USA; Boston VA Healthcare System, Boston, MA, USA; Sawyer Scientific, LLC, Boston, MA, USA; McGovern Institute for Brain Research, Massachusetts Institute of Technology, Cambridge, MA, USA; Brain and Mind Center, University of Sydney, Sydney, NSW, Australia

**Keywords:** fMRI, Dynamics, Attractor landscapes, Neuroscience, Bifurcations

## Abstract

Most human neuroscience research to date has focused on statistical approaches that describe stationary patterns of localized neural activity or blood flow. While these patterns are often interpreted in light of dynamic, information-processing concepts, the static, local, and inferential nature of the statistical approach makes it challenging to directly link neuroimaging results to plausible underlying neural mechanisms. Here, we argue that dynamical systems theory provides the crucial mechanistic framework for characterizing both the brain’s time-varying quality and its partial stability in the face of perturbations, and hence, that this perspective can have a profound impact on the interpretation of human neuroimaging results and their relationship with behavior. After briefly reviewing some key terminology, we identify three key ways in which neuroimaging analyses can embrace a dynamical systems perspective: by shifting from a local to a more global perspective, by focusing on dynamics instead of static snapshots of neural activity, and by embracing modeling approaches that map neural dynamics using “forward” models. Through this approach, we envisage ample opportunities for neuroimaging researchers to enrich their understanding of the dynamic neural mechanisms that support a wide array of brain functions, both in health and in the setting of psychopathology.

## Author Summary

The study of dynamical systems offers a powerful framework for interpreting neuroimaging data from a range of different contexts, however, as a field, we have yet to fully embrace the power of this approach. Here, we offer a brief overview of some key terms from the dynamical systems literature, and then highlight three ways in which neuroimaging studies can begin to embrace the dynamical systems approach: by shifting from local to global descriptions of activity, by moving from static to dynamic analyses, and by transitioning from descriptive to generative models of neural activity patterns.

## INTRODUCTION

Making sense of the inner workings of the human brain is a daunting task. Whole-brain neuroimaging represents a crucial device for reducing our uncertainty about how the brain works. But what if the assumptions inherent within traditional neuroimaging analyses have us on the wrong track? In many ways, neuroscience is relatively preparadigmatic (Kuhn, 1962), akin to the field of biology before the insights of Charles Darwin, or chemistry before atomic theory. With this in mind, how then should we approach modeling the brain? We suggest that a dynamical systems perspective provides a path for scientists to break out of the piecemeal progress circumscribed by traditional, static data-fitting statistical procedures. This modeling approach is also ideally suited to mechanistic accounts of the emergence of actions, emotions, and thoughts. We argue that dynamical systems theory (DST) is naturally suited to discussing the temporal aspects of neural and behavioral phenomena, as well as how interactions—within the brain and between the brain and external phenomena—unfold over time.

Since the cognitive revolution, neural processes have been routinely described in terms of manipulations of discrete “states,” “symbols,” or “codes” (Brette, 2019). The prevailing analogy used by this approach is the notion of “digital computing”: The brain is argued to “process information” by flexibly rearranging between different states. This approach naturally leads to a view of the brain as a mosaic of disjoint, independent functional units—consider the oversimplified conception of the amygdala as exclusively devoted to processing “fear” (Pessoa & Adolphs, 2010). This strategy has generated a “parts list” for neural processes, but only rarely pays close attention to how the parts *interact* in order to mediate the behavior of the system as a whole. Moreover the information-processing framework contains latent anthropomorphic thinking: coding, message-passing, and communication are metaphors that rely on the intuitive familiarity of social interactions—their neurobiological underpinnings are often left unstated (Brette, 2019).

In contrast to the view of the brain as a mosaic of quasi-independent functional units or agents, DST frames neural phenomena in terms of trajectories governed by coupled differential equations (Beurle, 1956; Caianiello, 1961; Corchs & Deco, 2004; Freeman, 1975; Griffith, 1963; Grossberg, 1967; Jirsa et al., 1994; Schoner & Kelso, 1988; Wilson & Cowan, 1972; Zeeman, 1973). These equations naturally lend themselves to causal and mechanistic interpretations, thereby cashing out anthropomorphic metaphors in terms of simpler biophysical processes such as excitation and inhibition. While the mathematical research behind DST has a long history, nonlinear dynamical systems exhibit behavior difficult to analyze without simulation. Advances in computational power have rendered DST much more tractable as a tool for neuroimaging (Breakspear, 2017; Cabral et al., 2014; Deco et al., 2009, 2011, 2013a, 2013b, 2015, 2021; Deco & Jirsa, 2012; Ghosh et al., 2008; Gollo et al., 2015; Hlinka & Coombes, 2012; Pillai & Jirsa, 2017; Sanz Perl et al., 2021; Shine et al., 2019a). Further, the DST modeling framework has enabled simulations of neural dynamics that are predictive and generative: simulated trajectories can be used to fit specific datasets (beim Graben et al., 2019; Golos et al., 2015; Hansen et al., 2015; Koppe et al., 2019; Vyas et al., 2020), but can also point researchers beyond data, for example, by contributing to experimental design and facilitating integration of findings from different paradigms and species.

An exhaustive survey of DST is beyond the scope of this review, but the key concepts have been described in depth in books accessible to neuroscientists (Durstewitz, 2017; Izhikevich, 2006; Rolls & Deco, 2010; Strogatz, 2015). Several neuroscience papers also serve as introductions to DST (Breakspear, 2017; Csete & Doyle, 2002; Favela, 2020, 2021; Miller, 2016; Shine et al., 2021), so here we will focus on how to integrate these modes of thinking with a functional, adaptive account of the brain. We will argue that DST is a lens that brings into sharp focus certain aspects of neural processing that are left somewhat blurred through the lens of the information-processing framework, including the importance of stability, flexibility, nonlinearity, and history dependence. Dynamical modes of description are particularly expressive for describing how humans and other animals pursue survival goals in ever-changing situations in ways that are both stable and fluid. More specifically, we argue that human neuroimaging, due to the availability of whole-brain sampling of brain dynamics, is especially suited to leverage concepts from DST (Deco et al., 2015; Galadí et al., 2021; Kringelbach & Deco, 2020). Importantly, beneath the surface-level complexity and abstraction of differential equations, DST enables a *visual* style of thinking that all neuroscientists can make use of in order to uncover causal and functional mechanisms (Daunizeau et al., 2012; Golos et al., 2015; Izhikevich, 2006; McIntosh & Jirsa, 2019; Rabinovich et al., 2006, 2015, 2020; Rabinovich & Varona, 2011; Shine et al., 2021; Wong & Wang, 2006).

In the first section of this review, we outline key concepts from DST that serve as building blocks for intuitive models of neural function. We then go on to suggest three ways in which current neuroimaging techniques can be productively combined with DST, thereby creating a powerful new vantage point from which to view the brain.

## A VIEW OF THE BRAIN THROUGH THE DYNAMICAL SYSTEMS PRISM

Traditional functional analyses of brain areas have allowed researchers to identify statistically reliable neural “puzzle pieces.” These methods give us insight into *what* a brain area or network may functionally mediate, but not *how* this mediation unfolds in time, or better yet, how coordinated interactions between the identified neural regions manifest as behavior. Our claim is that DST is the ideal framework for piecing together this brain-behavior puzzle, given that it foregrounds interaction and timing (McIntosh & Jirsa, 2019). Moreover, a dynamical systems perspective may suggest principled ways to reformulate psychiatric conceptions (Durstewitz et al., 2021) and “folk psychological” terms used to describe behavior, such as “attention,” “memory,” “emotion,” and “cognition,” and the functions of a given region may be better understood as integrated network-level trajectories rather than modular and localizable processes (Hommel et al., 2019). Conversely, the functions of some localized areas may be better conceived in terms of their effects on network dynamics, rather than in terms of psychological concepts.

**Figure 1.**
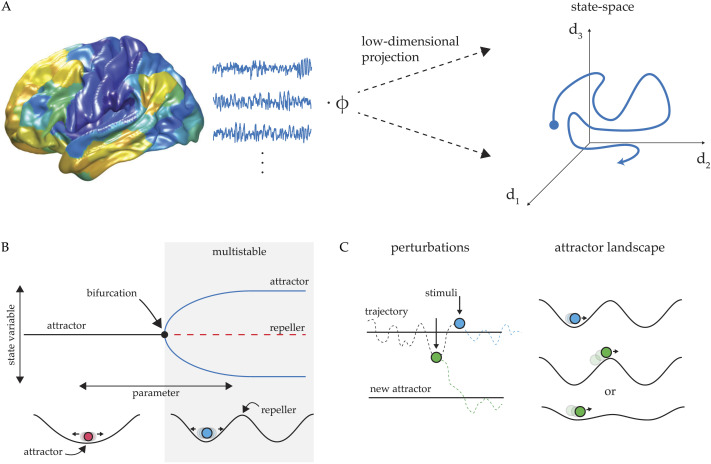
Overview of state space concept. (A) Large multivariate recordings of brain activity, such as in neuroimaging datasets, can be more tractable to analyze and visualize after first projecting the data into a state space sensitive to a desired feature in the data—for example, principal components for variance, or independent components for distinct signals. (B) Upper panel: pitch-fork bifurcation diagram showing a parameter change that transitions the system from a single stable attractor regime to a multistable regime with two stable attractors (blue lines) and one unstable attractor (red, dotted line). Lower panel: a potential energy landscape depiction of the same unistable and multistable regimes from above. (C) Identifying the attractor landscape of a system provides a reference for the system’s dynamics, which then predicts distinct response to perturbation. External input to a system can be treated either as a perturbation to the system’s trajectory or as a deformation of the system’s attractor landscape.

DST characterizes how a system—a neuron, a circuit, or even the whole brain—changes over time. A dynamical system is defined by its *state space* (or phase space), which characterizes the configurations available to the system. The dimensions of the state space specify the systems’ possible dynamics. For example, each dimension could be the firing rate of a neuron, or the metabolic activity of voxels, or the intensity of a stimulus. At any instant of time, the system is understood as occupying a *point* in its state space; a *trajectory* is a path through the state space, mapping how the values for each dimension change over time (Figure 1). *Differential equations* stipulate how the system’s trajectory will evolve over time from a chosen starting point (the *initial conditions*).

DST enables concise descriptions of families of trajectories that share qualitative properties. For example, if a family of trajectories all tend toward a particular region of state space, then that region is called an *attractor* (the simplest of which is called a *fixed point* attractor). The parts of state space from which the system finds itself “drawn” to an attractor forms the corresponding *basin of attraction*. The term “basin” here alludes to a valley in a mountain range — a ball placed on any slope of a valley will roll to the bottom. Understanding a state space as a landscape is an analogy that holds even in high-dimensional systems that cannot be visualized. The idea of an attractor provides an intuitive, mechanistic account of stability: a system in an attractor can be bumped or perturbed, but as long as the system stays within the attractor basin, it will eventually return to the bottom of the basin, like a marble rolling to the bottom of a bathtub. In contrast, a *repeller* is an inverted attractor, and therefore analogous to the top of a hill or a ridge: a system precariously balanced on a repeller.

The topography of fixed points isn’t always so clear cut. Indeed, fixed points can contain both attractive and repulsive properties, as is the case with a *saddle node*, which can be thought of topographically as similar to a mountain pass—unstable in one direction (i.e., you could just as easily move backward or forward along the path) but stable in another (i.e., it’s hard to climb the mountains on either side). Features such as saddle nodes inherently increase the potential complexity of emergent dynamics; however, it is important to point out that these qualitative features can only be identified when the differential equations of a system are posited. This implies that assigning terms such as “attractor” or “saddle” to a family of dynamic trajectories derived from data is necessarily dependent on the choice of model and cannot be inferred directly from data.

The set of all possible motivational states of an animal is an example of an *attractor landscape* (Deco & Jirsa, 2012; Shine, 2021) or “energy” landscape (though the use of the term “energy” is based on a mathematical analogy and need not possess the same physical dimensions as energy). The attractor basin of any given goal-oriented state must not be too deep: if an animal becomes so unwavering in its search for food that it is not perturbed by the appearance of a predator, then it is unlikely to survive for very long. Thus, behavioral flexibility requires that certain stimuli can nudge the system from one attractor basin to another. In other words, the trajectories of a flexible neural system are likely to traverse regions of state space that are repellers, since such regions are poised to enter nearby attractor basins. Another example of an attractor landscape is the space of perceptual targets that can capture attention (Rabinovich et al., 2013). Focused, unwavering attention on a target might correspond to the system being in a valley that is much deeper than neighboring ones, and from which the system cannot easily be dislodged by distractors. Similarly, high distractibility should correspond to a landscape of shallow attractors. Depending on the modeling goal, DST can be used to simulate how individual psychological constructs change over time (e.g., anger; Hoeksma et al., 2007), or how mental states shift across a landscape of multiple competing mental states, jostled by environmental forces (Jirsa & Kelso, 2004; Riley & Holden, 2012; Tognoli & Kelso, 2014). Beyond attractors, there are more subtle qualitative patterns, such as those associated with transient dynamics, that may be required to characterize trajectories exhibiting both recurring phases and variability or flexibility (Rabinovich et al., 2008; Rabinovich & Varona, 2011).

These external transient stimuli can be considered using the language of DST: for a system residing in state space, the only way for the system to move against the direction prescribed by the space is through a *perturbation*. In fact, determining whether a perturbation is considered “small,” or an attractor basin is considered “deep,” depends on their relative scales, as well as the exact position of the system within the attractor basin. For a system occupying the deepest point in a given attractor, perturbations below a certain scale will never push the system out of the attractor basin. If a system has already been perturbed so that it is near the ridge separating an attractor basin from that of an adjacent attractor, a relatively small push may be all that is needed to disrupt stability (Figure 1C). In the case of attention, this implies that, however focused an attentional state may be, there will be a distractor or combination of distractors that will have sufficient magnitude to push the system out of the corresponding attractor basin. Difficulties in maintaining attentional focus may arise from neural disruptions or developmental abnormalities that change the attractor depth of a target relative to the magnitude of perturbations, rendering attention easily captured by distractors (Duch, 2019; Iravani et al., 2021; John et al., 2018).

There are theoretical tools that motivate segmenting the brain into quasi-independent subsystems; we will now argue that this parcellation is far more illuminating than the traditional mosaic of functions. DST is not simply a taxonomy of attractors, repellers, and other qualitative features of trajectories. Important insights are derived from the study of *bifurcations*: qualitative changes to state space that arise from smooth *parameter* changes. Parameters, also referred to as “codimensions,” are distinct from the dimensions that define the state space. A typical example of a bifurcation is the transition from quiescence to stable repetitive spiking in the two-dimensional FitzHugh–Nagumo model and its descendants (FitzHugh, 1955; Izhikevich, 2006). In this simplification of the Hodgkin–Huxley model of the action potential, the excitatory input to the model neuron serves as a parameter, while the two dimensions are voltage and recovery, which characterize the spiking behavior. Increasing the input can trigger a “subcritical Hopf bifurcation,” in which a point attractor, the stable quiescent state, becomes unstable and an attractive *limit cycle* forms, such as is the case for periodic action potentials. As with all concepts in DST, bifurcations have a precise meaning only when we specify the model equations. But awareness of the general idea may point researchers toward mathematical models and theoretical insight. For example, in the case of the motivational attractor landscape discussed above, a bifurcation could occur if the environment affords only one salient goal initially, but affords two, say, eating and mating, after a transition arising from a parameter change, such as a decrease in perceived danger—the shift from one to two motivational attractors constitutes a bifurcation. Bifurcations have also been used to model the development of psychiatric disorders such as depression (Ramirez-Mahaluf et al., 2017).

## NEUROMODULATING THE MANIFOLD

What kinds of neural phenomena can deform the multidimensional attractor landscapes of the brain? Viewing neuromodulatory ligands such as dopamine, noradrenaline, and serotonin as *parameters* of subnetworks in the brain may provide fresh perspectives on how the brain flexibly alters its own low-dimensional neural dynamics. There is long-standing evidence that neuromodulatory tone is tightly coupled to cognitive function, often by way of an inverted U-shaped relationship (Arnsten, 1998)—for example, noradrenaline can transition an individual from a disengaged state to an engaged mindset back to disengaged. To test whether these capacities were linked to attractor landscape dynamics, Shine et al. (2018) mimicked the effects of neuromodulatory tone on neuronal activity by altering neural gain—effectively tuning how much influence individual populations in the network have over one another. Increasing neural gain at intermediate levels of excitability caused an abrupt, nonlinear increase in interregional synchrony that overlapped with empirical network topological signatures observed when analyzing task-based fMRI data (Shine et al., 2016). This same model was used to demonstrate a gain-mediated increase in interregional transfer entropy (Li et al., 2019). Given the similarity in the mechanisms by which neuromodulatory chemicals impact neural gain (Shine et al., 2021), we expect other neuromodulatory ligands to have similar effects on network dynamics, with idiosyncrasies that betray their unique functions (Kringelbach et al., 2020).

**Figure 2.**
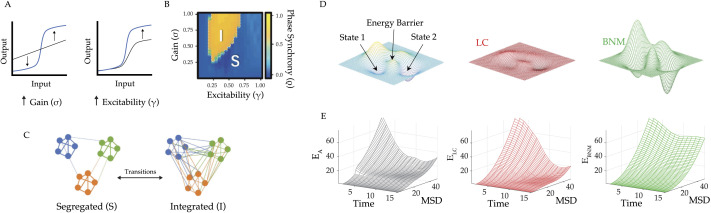
Neuromodulating the manifold. (A) Using a neural mass model implemented in The Virtual Brain, the input-output curve defining the activity of a slow variable was manipulated in two distinct ways: the sigmoid curve was steepened (left, neural gain) or amplified (right, excitability). (B) varying neural gain and excitability caused an abrupt switch in systems-level dynamics—by increasing neural gain, the system shifted from a Segregated state (“S,” low phase synchrony) into an Integrated state (“I,” high phase synchrony). (C) Schematic diagram of functional brain networks in the Segregated (i.e., “S”) and Integrated (i.e., “I”) phases—in the Integrated state, there are increased connections present between otherwise isolated modules. (D) Upper panel: an energy landscape, which defines the energy required to move between different brain states—by increasing response gain, noradrenaline is proposed to flatten the energy landscape (red); whereas by increasing multiplicative gain, acetylcholine should deepen the energy wells (green). Lower panel: empirical BOLD trajectory energies as a function of mean squared displacement (MSD) and sample time point (TR) of the baseline activity (black) and after phasic bursts in the locus coeruleus (a key noradrenergic hub in the brainstem, red) and the basal nucleus of Meynert (the major source of cortical acetylcholine, green)—relative to the baseline energy landscape phasic bursts in the locus coeruleus (red) lead to a flattening or reduction of the energy landscape, whereas peaks in the basal nucleus of Meynert (green) lead to a raising of the energy landscape. Panels A–C adapted from (Li et al., 2019) and Panels D–E adapted from (Munn et al., 2021).

Neuromodulatory ligands can also enact more subtle effects on state space dynamics (Figure 2). For instance, Munn et al. (2021) used a combination of 7T fMRI and statistical physics to demonstrate that the activity patterns in key hubs of the ascending arousal system differentially affect the brain’s attractor landscape. Specifically, activity in the locus coeruleus (the primary source of noradrenaline for the brain) was found to precede a flattening of the attractor landscape and hence allowed the system to leave an attractor with a smaller perturbation than was previously necessary. In contrast, blood flow in the basal nucleus of Meynert (the primary source of cholinergic inputs to the cortex) was found to precede moments in which the brain remained “stuck” in a deep well with a greatly diminished ability to escape. Importantly, these changes are also tied to alterations in phenomenological states. By analyzing fMRI data obtained during breath awareness meditation, Munn and colleagues found similar attractor landscape dynamics linked to alterations in internal awareness—specifically, the moments when meditators noticed that their thoughts had “wandered” from their breath. This phenomenon is also highly reminiscent of the notion of a noradrenaline-mediated “network reset” (Sara & Bouret, 2012), which has also been used to explain switches in perceptual stability associated with bistable images (Einhäuser et al., 2008), and hence may represent a fundamental feature of the intersection between neuromodulatory tone and network-level dynamics.

## DYNAMICAL SYSTEMS THEORY FOR HUMAN NEUROIMAGING

**Figure 3.**
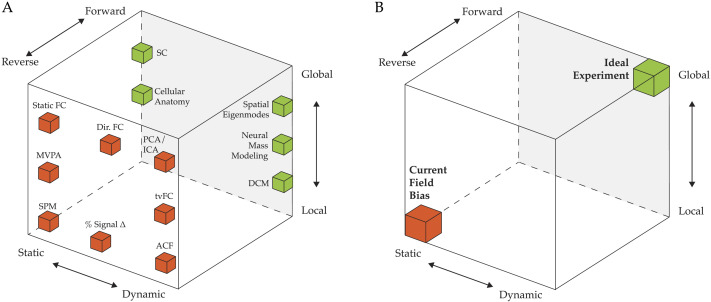
The space of analytic approaches in human neuroimaging. A nonexhaustive collection of different popular methods for analyzing human neuroimaging data, embedded into a cube axes that highlight three key dynamical systems characteristics: Static-to-Dynamic (*x*), Reverse-to-Forwards (*y*), and Local-to-Global (*z*). We have argued that embracing the dynamical systems perspective requires moving to the top right of the cube (i.e., the “Ideal Experiment”). While the theoretical goal of models should be dynamic, global, and built with forward modeling in mind, multiple approaches are necessary for comprehensive understanding, especially the analysis of empirically obtained data (the reverse approach). For further clarity, methods with high loading on the “Reverse” axis are colored red, and those high on the “Forwards” axis are colored green. Note that some methods cover larger portions of this space than has been designated here (e.g., both PCA and ICA can be used in either a dynamic or a static sense) and that the boxes should not be considered as strong limits for particular methods, but rather as an approximate consensus for how particular methods are currently used by the majority of neuroimaging studies in the field. SPM = statistical parametric mapping; FC = functional connectivity; MVPA = multivoxel pattern analysis; tvFC = time-varying functional connectivity; Dir. FC = directed functional connectivity; PCA = principal components analysis; ICA = independent components analysis; ACF = autocorrelation function; DCM = dynamic causal modeling; SC = structural connectivity.

Reframing neuroimaging data in the language of DST offers an exciting opportunity to investigate the brain using a precise language tailor-made for describing the distributed, dynamic, and highly integrated nature of the brain. Following in the footsteps of pioneering studies in the field that combined neuroimaging, computational modeling, and cognitive neuroscience tasks to advance our understanding of the rules that govern dynamical activity in the brain (Box 1), we identify three key principles through which neuroimaging researchers can adopt a dynamical systems perspective: zooming out from the local to the global level, trading off static for more dynamic descriptions of the brain, and moving from description to simulation (Figure 3). By designing neuroimaging approaches that embrace each of these aspects, we hope to entice the field toward more “ideal” experiments that will both expose the inner workings of the brain, but also identify more sensitive means for interacting with the complex, adaptive, and dynamic nature of the brain.

**Box 1.** A spectrum of dynamical systems approaches in neuroimaging Differential equations are becoming increasingly popular in DST modeling of neuroimaging data (beim Graben et al., 2019; Kringelbach & Deco, 2020; Wang et al., 2019). However, as in the case of data-oriented modeling techniques represented schematically in Figure 1, differential equation-based methods occupy a continuous “feature space of models,” not all of which use the full suite of DST concepts. Three key features have helped us make sense of the ever-expanding literature on dynamical modeling and DST: (1) the extent of focus on qualitative or mechanistic explanations using qualitative patterns like attractors and bifurcations, (2) the extent of focus on quantitative fitting of data, and (3) the degree to which characterization of data is employed to explain behavior (cognition, emotion, and other processes).

While it is tempting to view qualitative and quantitative modeling as mutually exclusive extremes on a continuum, it is possible for a single model to excel at both. Recent work demonstrates that close attention to data and precise mechanistic models can go hand in hand (Breakspear, 2017; Deco & Jirsa, 2012; Kringelbach & Deco, 2020; Shine et al., 2021; Wang et al., 2019). Nevertheless, the sheer complexity of data, as well as the plurality of research goals, means that there cannot be a “one-size-fits-all” approach to dynamical modeling of the brain. Ideally, models that perform quantitative fitting and those that focus more on qualitative characterization can mutually constrain and inspire each other.

The third highlighted feature of DST models—the mapping between brain dynamics and behavior—in our view has the most scope for growth. Given the complexity of the brain, it is natural to treat it as a phenomenon on its own, rather than a central part of a wider set of behavioral phenomena: cognition, emotion, and action. Given that these phenomena can themselves be described in terms of dynamics, a key goal of DST in neuroimaging must be to show, beyond mere correlation, how specific patterns of neural dynamics give rise to specific patterns of *behavioral* dynamics. In other words, the neuroimaging field will benefit from DST models that not only generate accurate simulations and interface with lower level neural mechanisms, but also provide a causal and functional account of the dynamics of emotions or broad cognitive modes. Early steps in this direction include studies of meditation and sleep that map DST concepts directly onto neuroimaging data (Deco et al., 2019; Galadí et al., 2021; Melnychuk et al., 2018; Munn et al., 2021). Neuroimaging studies of clinical and psychiatric conditions are beginning to be viewed through the DST lens, including epilepsy (McIntosh & Jirsa, 2019), migraine (Dahlem & Isele, 2013), and schizophrenia (Loh et al., 2007). There are many opportunities for close integration between DST as a way to study neuroimaging data and DST as a perspective on how symptoms are generated, such as in attention deficit hyperactivity disorder (Iravani et al., 2021), autism (Duch, 2019), and depression (Ramirez-Mahaluf et al., 2017).

### Zooming Out to View the Whole Network

The popular “massively univariate” statistical parametric mapping (SPM; Figure 3) approach employed in most fMRI research precludes a deep understanding of the dynamic brain, with its interconnections influencing each other and changing over time. In this traditional approach, following careful preprocessing steps (Esteban et al., 2019), independent statistical models are fit to a behavioral task paradigm (convolved with a hemodynamic response function or finite impulse response model to account for hemodynamic delay) to the time course of either a single voxel or an averaged, summary time series calculated from a (hopefully predefined) region of interest. Such approaches have been successful in identifying regions with particular functions (such as the fusiform face area), via the clustering of voxels independently identified with statistical models that typically involve task contrasts (such as activation during face vs. scene viewing). The early success of these methods has entrenched a relatively static mindset among academics that hinders more detailed explanations involving multiple regions interacting over time. While there are numerous examples of pioneering work examining whole-brain neuroimaging with circuits-level explanations, we maintain that purely stationary statistical models are insufficient for a mechanistic understanding of cognitive phenomena in both healthy and diseased states.

In contrast, the DST approach has an inherent and direct link to underlying mechanisms. For example, instead of performing a univariate analysis and reporting that a face viewing task “activates” the fusiform face area, researchers could report how the entire brain activation patterns shift from one state (while viewing scenes) to another (while viewing faces) and back again over time. Even with a univariate analysis, this perspective could be supported by routinely including animation of fMRI activity, and by using unthresholded surface maps for improved visualization. Multiecho sequences may even allow for sufficient denoising (Kundu et al., 2017) to examine individual trials, precluding the need for the trial averaging that occludes network states influencing activation movements. Unthresholded animation, especially denoised, could then hint at a trajectory between states. Crucially, this approach would then offer additional steps, such as interrogating the likely neural processes that could have caused the differences between cognitive capacities (assuming a good observational model), or prediction of how the dynamics should change, given an intervention such as transcranial magnetic stimulation or a suitably chosen pharmacological agent.

Multivariate analyses have been steadily growing in popularity over recent years. These approaches begin with the assumption that neural representations are nonlocal: that is, that the functional capacities of the brain rely on distributed patterns of activity that reflect the influences that neural regions have over one another. The most widely adopted reverse (i.e., data fitting) multivariate approaches for measuring these effects are functional connectivity fMRI (FC), seed-based and independent component analysis (ICA), multivoxel pattern analysis (MVPA), and the effective connectivity approaches of psychophysiological interactions (PPI) and Granger causality (Figure 3). These methods provide insight into systems-level brain organization: for instance, the idea of a set of modular communities (derived using functional connectivity) that loosely relate to distinct functional capacities (Smith et al., 2009). However, despite this clarity, it is important to note that these methods are still primarily focused on fitting data rather than creating a generative model. As such, a substantial theoretical gap still remains between the appearance of these patterns and the mechanistic processes that could give rise to them. As we mentioned above, this problem can be mitigated in large part by grounding our investigations of neuroimaging data in a dynamical systems framework.

Other popular methods are based on the justified assumption that neural activity is low dimensional: the inherent degrees of freedom of neuroimaging data are typically far fewer than the number of different recordings that sample the brain (Churchland et al., 2012; Durstewitz, 2017; Gallego et al., 2020; Gotts et al., 2020; Shine et al., 2019a, 2019b). Embracing this assumption—using popular approaches such as principal component analysis (PCA) and ICA (Figure 3)—means that experimenters can reduce the number of independent variables that they need to track, a process that makes both interpretation and modeling substantially easier. In neuroimaging, the goal is typically to reduce the dimensionality of voxels or electrodes such that what was once an unwieldy dataset can now be effectively tracked (and visualized) in low-dimensional (“state”) space. In a recent fMRI study, Shine et al. (2019a) used PCA to reduce regional BOLD activity across multiple tasks to a set of low-dimensional components that were then shown to link clearly to analyses based on cognitive neuroscience, network neuroscience, DST, and neuromodulatory receptor expression. Crucially, certain critical assumptions of the dimensionality reduction approach are incompatible with aggressive preprocessing steps often used to “clean” data (Gotts et al., 2020)—careful modeling clearly shows that these strategies often “throw out the baby with the bathwater,” and hence should be applied with abundant caution. Regardless, this approach only scratches the surface of the potential for dimensionality reduction in systems neuroscience, as evidenced by the many examples from nonhuman studies (Chaudhuri et al., 2019; Mastrogiuseppe & Ostojic, 2018; Stringer et al., 2016).

Graph theory provides another means for embracing the distributed nature of neural activity patterns (Sporns, 2015), enabling a more harmonious integration with DST. One such approach treats regions of the brain as nodes of a network (or graph), and then defines the edges between these nodes according to the strength of temporal similarity (for instance, using a Pearson’s correlation or wavelet coherence). Following this step, mathematical tools (Fornito et al., 2016) can be used to infer topological properties of the network, that is, those features that are present in the data, irrespective of the specific implementation (Sporns, 2013), and how these properties change as a function of factors such as the cognitive demands of the task (Shine & Poldrack, 2018). The approach is not without pitfalls, as seemingly trivial choices (such as the presence and extent of edge thresholding) can have substantial impacts on the conclusions inferred about particular cognitive capacities (Hallquist & Hillary, 2019). In addition, there is also evidence that the ability to decipher stable nodes can vary substantially as a function of different cognitive tasks (Salehi et al., 2020). Despite these concerns, these approaches do reveal important aspects of the systems-level dynamics of the brain, and hence are capable of generating predictions about how neural activity is grounded in the underlying neurobiology. Two pertinent examples from recent work involve linking brain network integration to the diffuse projections of the ascending noradrenergic system (Munn et al., 2021; Shine et al., 2016, 2018) and the matrix regions of the thalamus (Müller et al., 2020a, 2020b).

### Shifting From Static to Dynamic

An organism is a constantly changing web of biophysical and electrochemical interactions. A natural consequence of this organization is that the manner in which stimuli are processed depends on the state of the organism at the precise moment that a stimulus arrives. In other words, the brain is inherently dynamic, and cannot be understood with mere static descriptions. For instance, it is essential to examine not only how activity levels in voxels change over time, but also to model how voxels influence each other. Unfortunately, the majority of approaches used in modern neuroimaging contain a hidden assumption of stationarity—when viewed through the lens of DST, this amounts to assuming that the brain is always in the same position in state space when a stimulus arrives, which is difficult to justify.

One simple way to incorporate dynamics into modern neuroimaging approaches is to extend analyses beyond the typical assumptions of zero-lag correlation that permeate the field. These patterns are not uninterpretable in their own right—for example, the robustness and relative invariance of static network parcellations derived from long fc-fMRI scans suggests a form of slow dynamic stability, rather than an artifact of averaging. However, there is also evidence that, by calculating functional connectivity patterns across an entire scan, investigators potentially average across reconfigurations that occur over shorter time scales (Faskowitz et al., 2020; Honey et al., 2007; Karahanoğlu & Van De Ville, 2015). Fortunately, methods exist to soften these constraints (Robinson et al., 2021). For instance, tracking time-shifted correlations in fMRI showed that the well-known zero-lag temporal correlation structure of intrinsic activity emerges as a consequence of neural trajectories, assessed by their lag structure (Mitra et al., 2015) (Figure 3). At their extreme, these patterns can be interpreted as spatiotemporal traveling waves (Raut et al., 2021) or eigenmodes (Robinson et al., 2021), which are amenable to dynamical systems modeling (Koch, 2021). Traveling wave models are an example of a broad class of coarse-graining approaches in DST that include neural field, neural mass and mean field models (Bojak et al., 2010; Byrne et al., 2020; Deco et al., 2013b; Müller et al., 2020a; Shine et al., 2021; Wang et al., 2019). Another pertinent example comes from the field of time-varying functional connectivity, which typically breaks a standard neuroimaging scan into smaller windows and then characterizes fluctuations in correlation patterns over time (Lurie et al., 2020). In both cases, embracing the dynamics inherent in interregional coordination can pave the way to more powerful generative models of the human brain and its mediation of behavior.

A common criticism of fMRI is that the typical temporal resolution is slower than the time scales of most perceptual and behavioral changes. While this is true for fast behavioral choices, homeostatic processes in humans and other organisms necessarily take place at a variety of temporal scales. The fastest perceptions and reactions are embedded in slow dynamical trajectories that may correspond to phenomena such as mood, affect, or cognitive mode, which in turn are embedded in even slower trajectories such as hormonal/circadian rhythms and so on. The temporally and spatially coarse grained nature of whole-brain functional imaging make it well suited to characterizing “quasi-invariants”—neural contexts within which perception, thinking, and action are framed. Neural dynamics is organized across an intertwined temporal hierarchy, with causal relationships operating in both directions. For example, slower oscillations modulate fast oscillation (Tort et al., 2010), and, psychologically, a sudden fright may cause a lasting change of mood. As a first approximation, it is useful to think of slower fMRI findings as a window into slow processes that set the context for faster processing. Further, clever task designs can identify faster responses, on the order of hundreds of milliseconds (Lewis et al., 2018), so even faster dynamics can be studied.

Another potential barrier to application of dynamical analysis of fMRI is the fact that most fMRI paradigms involve analysis of data from predetermined epochs, whether they are blocks of stimuli or collections of rapidly presented events. While traditionally considered important for ensuring effective signal-to-noise properties, the constraints imposed by these approaches can limit the conclusions made about the dynamical processes at play. Moreover, a pure task-based division of neural recordings will average out any functional variability that is independent of the task structure. In other words, the underlying assumption is that all functionally relevant neural dynamics are strongly correlated to the temporal division assumed by the experimenter. Fortunately, newer task structures such as movie watching (Finn & Bandettini, 2020; Meer et al., 2020) and videogames (Richlan et al., 2018) do not impose the event structures that are typically used in signal-averaging approaches. Instead, dynamical models can be constructed that predict how the trajectory of brain states will change in concert with the videogame, and these simulations can then be compared with the fMRI data acquired.

The notion of attractor landscapes provides enticing links to whole-brain neuroimaging and suggests a set of neural trajectories that can be applied to neuroimaging data. In this framing, brain states evolve along the attractor landscape topography, much like a ball rolls under the influence of gravity down a valley and requires energy to traverse up a hill, this corresponds to an evolution toward an attractive or repulsive brain state, respectively. This technique can resolve what might otherwise be obscured states of attraction (and repulsion) in a multistable system and has been successfully applied to the dynamics of spiking neurons (Tkačik et al., 2015), BOLD fMRI (Munn et al., 2021; Watanabe et al., 2013, 2014), and MEG (Krzemiński et al., 2020). The approach offers several conceptual advances, but perhaps most importantly, it renders the otherwise daunting task of systems-level interpretation relatively intuitive. Importantly, this framework extends beyond mere analogy, as the topography of the attractor landscape shares a 1-to-1 correspondence with the generative equations required to synthesize realistic neural time series data (Breakspear, 2017). For example, Munn et al. (2021) compared trajectories of BOLD activity following phasic bursts of subcortical regions of the ascending arousal system, and by leveraging the attractor landscape approach it was apparent adrenergic and cholinergic neuromodulation actively modulated the strength of an attractor state.

### Moving From Description to Simulation

All computational models in biology can be situated on a continuum from “reverse” to “forward,” based on their relationship with experimental data (Gunawardena, 2014). Statistical models proceed in the “reverse” direction: the modeling begins with experimental data and then “reverse engineers” the causal mechanisms that generated the data. In contrast, “forward” modeling starts with known or hypothetical causal mechanisms, which are used to generate patterns that mirror key aspects of experimental data (Breakspear, 2017). These two approaches were combined in what is arguably the most successful model in neuroscience, the Hodgkin–Huxley model of action potential generation (Hodgkin & Huxley, 1952): the data fitting facilitated the discovery of a system of differential equations that pointed toward the mechanisms underlying action potential generation.

At scales larger than the single neuron, forward modeling becomes increasingly underconstrained by experimental data. There is also no consensus on the neurobiological underpinnings of neuroimaging techniques (Breakspear, 2017). But the lack of constraint by data does not mean that forward models cannot be built: careful analysis of anatomy, behavior, and evolutionary history can provide modelers with well-justified mechanisms that can be captured by differential equations. Further, given the variability of neural and behavioral data, it does not make sense for generative models to cleave too closely to specific quantitative recordings. Qualitative descriptions and predictions can be more robust than quantitative data fits, as they generalize more easily, being less sensitive to idiosyncratic features of specific experiments. For instance, the notion that acetylcholine and noradrenaline can modulate attractor landscape topography (Munn et al., 2021) can be imported into the design of future experiments, not only in the context of meditation, but also to attention more broadly construed. It also creates bridges with nonhuman research techniques that can directly manipulate these neuromodulators.

There are existing software programs for simulating dynamical systems, such as the Brain Dynamics Toolbox (Breakspear & Heitmann, 2010; Heitmann & Breakspear, 2018) and the Virtual Brain (Ritter et al., 2013; Sanz-Leon et al., 2015; Schirner et al., 2021; Spiegler et al., 2016). Using these tools, DST concepts can be directly tested through comparison of model outputs with fMRI data. However, because the field of DST in neuroimaging is rapidly evolving, software packages may be less flexible than custom simulations written in programming languages like *MATLAB*, *Python*, or *Julia*. For example, custom code can be used to construct layer-specific models that incorporate the precise, compartment-specific connectivity principles that are present in the cerebral cortex (Braitenberg & Lauria, 1960; Du et al., 2012; Havlicek & Uludağ, 2020; Stephan et al., 2019). Regardless of the computational approach taken, the activity dynamics for each of the regions or neurons can be simulated, and the activity can then be convolved with a canonical hemodynamic response function, or better yet, with more advanced models of hemodynamics (Aquino et al., 2012; Pang et al., 2016). The output of this simulation can then be compared qualitatively with fMRI data collected during an experiment, with further iterations of the model bringing theory into closer contact with empirical data. This approach will be particularly powerful when combined with advances in fast sampling-rate (Polimeni & Lewis, 2021) and layer-resolved fMRI recordings (Huber et al., 2021; Polimeni et al., 2010), both of which will increase the precision with which models can be integrated with neuroimaging data.

It is important to note that a key constraint imposed by computational models is the degree of their abstraction from the “veridical”—the vast dimensionality of the adult human brain is undoubtedly more complex than a typical neural model can realistically simulate, such that even the most detailed computational model will likely lack the degrees of freedom to effectively characterize the true nature of the dynamical system with sufficient clarity and robustness. One way to mitigate this issue is to design modeling architectures to express a particular feature of neuroanatomy, and then, after investigating any interesting implications of the feature, compare the outputs of the model with empirical neuronal recordings. The Virtual Brain (Ritter et al., 2013; Sanz-Leon et al., 2015; Schirner et al., 2021) is an excellent example of a toolbox that affords access to this approach, and has been used to demonstrate important links between structure and function across many spatiotemporal scales. In these approaches, users define the network structure and computational model of interest, and then manipulate whichever parameters are of experimental interest. A complementary approach is to design more bespoke neural architectures, such as those that embrace interactions between the cerebral cortex and thalamus, and then work to determine what the benefits and costs of such an architecture might be. For instance, the presence of a population of relatively diffuse thalamocortical projections (as is the case for matrix thalamic nuclei; Jones, 2001; Müller et al., 2020a); can shift a network of corticothalamic neural masses into a quasi-critical regime characterized by the continual formation and dissolution of neuronal ensembles in such a way that maximizes a trade-off between network integration and segregation (Müller et al., 2020b). Although these approaches can be quite insightful, it is important to remember to pick a scale of modeling that matches both the mechanism of interest, and the particular imaging technique that the researcher is interested in interrogating.

A point worth stressing is that DST goes beyond the use of differential equations to fit data. For example, some variations of DCM (Cao et al., 2019; Friston et al., 2019) focus on data fitting but do not employ qualitative concepts such as attractor landscapes, limit cycles, or bifurcations, partly because they restrict themselves to the linear domain (Sadeghi et al., 2020), whereas more sophisticated nonlinear variations do (Daunizeau et al., 2012; Roberts et al., 2017a, 2017b). Models based on differential equations, whether linear or nonlinear, are also generative, and can simulate hypothetical BOLD data. In addition to the capacity for quantitative fits and simulations, DST offers conceptual tools that create bridges between data and neural mechanisms. In principle, any neuroimaging outcome measure can be generated by a well-designed forward model, but measures that embrace the complex, dynamical features of biological data (Bizzarri et al., 2019; Juarrero, 2002) will likely lead to a more rich causal understanding. Further, as we have mentioned at various points in this manuscript, the qualitative tools of DST—attractors, bifurcations, metastability, etc.—not only help account for data and neural processes, but also create natural links with the dynamics of behavior and cognition (also see Box 1).

## CONCLUSIONS

In this Review, we have argued that the DST framework has the potential to revolutionize the analysis of neuroimaging data and how this data accounts for behavior, both in artificial task-based protocols and more naturalistic situations such as movie watching. We have argued that embracing this perspective will enable the discovery of otherwise latent links between neural mechanisms and the patterns that we measure from standard imaging approaches, which in turn can be used to rapidly augment our understanding of the brain, both in health and disease. For instance, we argue that a renewed focus on time-varying dynamics via the identification of qualitative but well-characterized dynamical phenomena (such as stability and limit cycles) or ideally, the geometric or visual interpretation of results (e.g., in terms of attractor basins or saddles) emergent in whole-brain neuroimaging data, will lead to rapid progress in systems neuroscience. This paradigm shift is already well underway, as evidenced by numerous papers that have used neuroimaging to derive measures of stability, entropy, and low-dimensional attractor manifolds as a function of different task contexts (Chaudhuri et al., 2019; Koppe et al., 2019; Müller et al., 2020b; Munn et al., 2021).

There is much work to be done. Fortunately, a major benefit of the DST approach is that there exists a large corpus of fMRI data that can be reanalyzed within the frame imposed by dynamical systems, potentially leading to major new insights into the brain bases of higher order mental phenomena. To this end, we strongly recommend that interested neuroscientists reach out to and actively collaborate with computational modelers in order to build models that can make predictions and build deeper intuition and explanation for the data already acquired. Of course, the advent of higher spatial and temporal resolution data, and interventional datasets like those that combine optogenetics with fMRI (Ryali et al., 2016), will undoubtedly further accelerate progress. Nonlinear dynamical systems must be simulated, so advances in computational power fuel advances in what can be understood with DST. The synergistic interactions that will emerge between DST and imaging are a crucial step toward the maturation of the field of systems neuroscience.

## AUTHOR CONTRIBUTIONS

Yohan J. John: Conceptualization; Writing – original draft; Writing – review & editing. Kayle S. Sawyer: Conceptualization; Writing – original draft; Writing – review & editing. Karthik Srinivasan: Conceptualization; Writing – original draft; Writing – review & editing. Eli J. Müller: Conceptualization; Visualization; Writing – original draft; Writing – review & editing. Brandon R. Munn: Conceptualization; Writing – original draft; Writing – review & editing. James Shine: Conceptualization; Visualization; Writing – original draft; Writing – review & editing.

## FUNDING INFORMATION

James Shine, National Health and Medical Research Council (https://dx.doi.org/10.13039/501100000925), Award ID: 1193857.

## TECHNICAL TERMS

State space: A representation of all possible states that can be attained by the system (i.e., a point in state space).
Trajectory: The time course of a system given a particular set of initial conditions.
Attractor: A region of one or more fixed points that trajectories move towards.
Fixed point: A point in state space where the system is stationary (i.e., the derivative with respect to time is zero).
Basin of attraction: An area of state space from which systems will evolve towards a particular attractor.
Repeller: A region of one or more fixed points that trajectories move away from.
Attractor landscape: A state space containing multiple basins of attraction.
Perturbation: A small extrinsic change in the position of the system in state space (not governed by the system’s differential equations).
Bifurcation: A qualitative change in the behavior of the system produced by a change in a parameter of the differential equations.
Limit cycle: A region of state space that takes the form of a closed, cyclic trajectory.
